# Myalgic Encephalomyelitis (ME) or What? An Operational Definition

**DOI:** 10.3390/diagnostics8030064

**Published:** 2018-09-08

**Authors:** Frank Twisk

**Affiliations:** ME-de-Patiënten Foundation, Zonnedauw 15, 1906 HB Limmen, The Netherlands; frank.twisk@hetnet.nl; Tel.: +31-72-505-4775

**Keywords:** myalgic encephalomyelitis, chronic fatigue syndrome, diagnosis, neuromuscular, neurological disorders

## Abstract

Myalgic encephalomyelitis (ME), identified as a new clinical entity with distinctive features in 1956, was originally considered as a neuromuscular disease. In 1988 the Centers for Disease Control and Prevention introduced the ill-defined concept of chronic fatigue syndrome (CFS). As predicted, CFS, unjustly considered to be a synonym for ME, pushed ME to the background. To develop effective therapies for of ME and CFS, it is essential to investigate patients with ME specifically. For that reason, an operational definition of ME is indispensable. This article proposes an operational definition based on the most recent formal definitions and symptoms observed in ME. ME is a multi-systemic illness, which (1) often has a sudden onset, in most cases a respiratory and/or gastro-intestinal infection, but a gradual or more dramatic onset is also possible; (2) has an epidemic and an endemic form; (3) has an unique clinical pattern deviating from other post-viral states; (4) is distinguished by muscle fatigability/prolonged muscle weakness after trivial exertion; (5) is accompanied by symptoms relating to neurological disturbance, especially of cognitive, autonomic, and sensory functions; (6) can be accompanied by symptoms associated with cardiac and other systems; (7) is characterized by fluctuation of symptoms (within and between “episodes”); (8) has a prolonged relapsing course; and (9) has a tendency to become chronic. In conclusion, a discriminative definition for ME contains four mandatory elements: (1) muscle fatigability/post-exertional muscle weakness lasting for days; (2) operational criteria for “neurological disturbance, especially of cognitive, autonomic and sensory functions”; (3) fluctuation of symptoms; and (4) a prolonged relapsing course. This tentative definition of ME justifies the qualification “neuromuscular disease”.

## 1. Introduction

Myalgic encephalomyelitis (ME) [1,2,3,4], in the literature also referred to as epidemic neuromyasthenia [5] and non-paralytic [3] or “atypical” [6] poliomyelitis, is a neuromuscular disease which was identified as a distinct clinical entity for the first time in 1956 [7]. At that time it was evident that muscular symptoms (e.g., paresis and myalgia) and neurological dysfunction were the hallmark features of ME [7]. ME [1,2,3,4] has been classified as a neurological disease by the World Health Organization (WHO) since 1969 [8,9].

In 1988 the US Centers for Disease Control and Prevention (CDC) introduced the ill-defined notion of chronic fatigue syndrome (CFS) [10]. CFS was redefined in 1994 [11]. The only mandatory feature of CFS [11] is (unexplained) chronic fatigue. Chronic fatigue must be accompanied by at least four out of a list of eight “minor” symptoms: sore throat; tender cervical or axillary lymph nodes; muscle pain, multi-joint pain without joint swelling or redness; headaches of a new type, pattern, or severity; unrefreshing sleep; and post-exertional malaise lasting more than 24 h [11]. The case criteria for CFS [11] define a heterogeneous group of patients with chronic fatigue (accompanied by 163 different combinations of “minor” symptoms) [12,13].

ME [1,2,3,4] and CFS [11] are two completely different concepts [14]. Chronic fatigue has never been described as a distinctive feature of ME [1,2,3,4], while muscle fatigability/prolonged post-exertional muscle weakness and specific neurological symptoms, discriminative features of ME [1,2,3,4], are not required to meet the diagnosis for CFS [11].

Despite the fact that ME [1,2,3,4], a typical neuromuscular disease, and CFS [11], a fatigue syndrome, are two completely different clinical entities by definition [14], CFS [11] has pushed ME completely to the background over the last decades. Thus, the vast majority of studies into “ME/CFS” published since 1990 relate to patients fulfilling the case criteria for CFS [11]. The confusion with regard with diagnosis and abnormalities which has arisen as a result of the introduction of the diagnosis of CFS was forecasted in 1988 by the renowned ME expert Dr. Betty Dowsett, who stated “The introduction of ‘chronic fatigue syndrome’ to designate ME does nothing to indicate the unique epidemio-logical, geographical, clinical, and laboratory findings in ME and can only add to the confusion surrounding the diagnosis, therapy, and prognosis of the condition” [2].

Due to the replacement of ME [1,2,3,4] by CFS [11], one of the last important studies investigating ME patients dates back to 1990 [3], just prior to the death of Melvin Ramsay, the recognized authority in ME. Dr. Ramsay was a consultant physician in infectious diseases at the Royal Free Hospital in London (United Kingdom) during an outbreak of ME in 1955 [15]. From that date until his death Ramsay was closely involved in ME cases.

To unravel the aetiology and pathophysiology and to develop effective therapies for ME [1,2,3,4], it is essential to study patients with ME as described in the medical literature. For that reason an operational definition of ME [1,2,3,4] is indispensable. 

The aim of this study was to compose an operational definition of ME based on the definition and description in the most recent relevant articles [3,4], the symptoms and signs observed in the various epidemics (1934–1983) and endemic cases of ME [1], and the symptoms often and less-often experienced by 420 patients with ME [3].

## 2. Method

**Figure 1:**
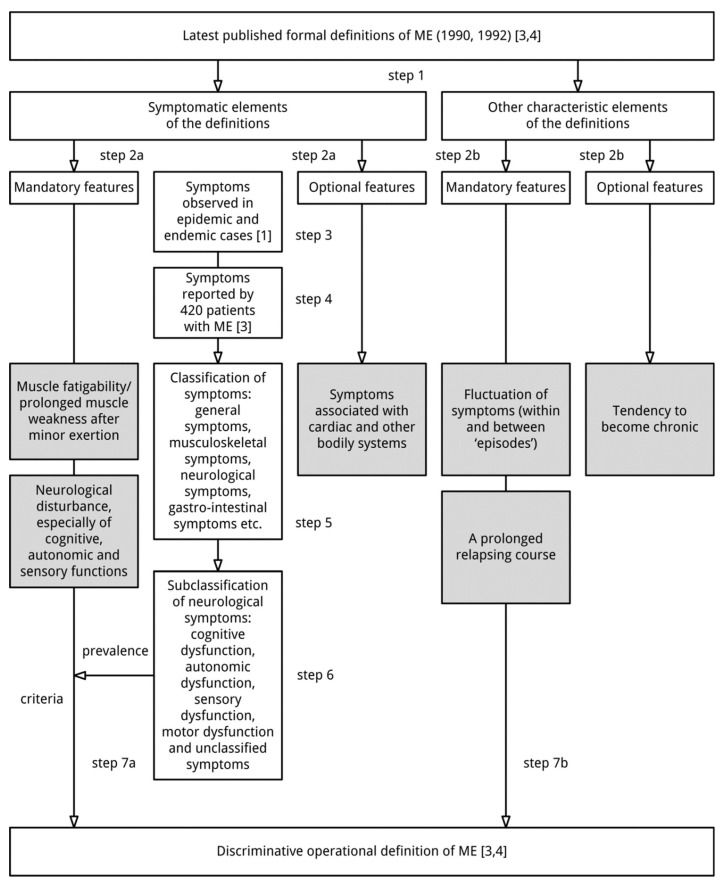
Method.

The starting point of the search of an operational definition of ME are the last two formal descriptions of ME [3,4]. As can be seen from Table 1 and Table 2, the definitions of ME in these two publications [3,4] are essentially the same. The elements were classified as symptomatic features or characteristic features of the disease (Figure 1, Step 1). Based on the text, elements were classified as mandatory or optional (Step 2). For example the phrase “a tendency to chronicity” [4] implies it does not apply to all patients: it is an optional feature. This also applies to “variable involvement of cardiac and other systems” [3].

The symptoms mentioned in epidemic and endemic cases of ME [1] were analysed and classified (Steps 3 and 5). The symptoms and symptom categories were checked against symptoms observed in 420 patients with well-defined ME [3] (Step 4).

In Step 2a it was learnt that in addition to muscle fatigability/prolonged muscle weakness after exertion, “neurological disturbance, especially of cognitive, autonomic and sensory functions” is a mandatory symptomatic feature in the definition of ME.

To define operational criteria, the 50 neurological symptoms mentioned in cases of ME [1,3] were divided into five categories (Step 6): cognitive dysfunction, autonomic dysfunction, sensory dysfunction, motor dysfunction, and unclassified symptoms.

The basic assumption was that a patient should experience at least one symptom related to cognitive dysfunction, at least one symptom related to autonomic dysfunction, and at least one symptom associated with sensory dysfunction in order to meet the operational criteria of ME. However, considering the frequency of the symptoms reported by 420 patients with ME [1,3] and the number of times specific symptoms were mentioned in the description of cases of ME [16], two changes were made (Step 7a). First, since only three symptoms observed [1,3] relate to cognitive disturbance, and not all patients experience these three symptoms [3], it was decided that “cognitive dysfunction” symptoms should be combined with three other symptoms often reported by patients with ME [3] (two unclassified neurological symptoms related to sleep and headaches) in order to meet the first operational criterion for “neurological disturbance”. Second, because symptoms related to sensory dysfunction are diverse and this category contains various symptoms often experienced by patients [1,3], it was decided that a patient should report at least two symptoms of the “sensory symptoms” subcategory to meet the minimal requirement for “sensory dysfunction”.

## 3. Results

### 3.1. Characteristics

#### 3.1.1. An Epidemic and an Endemic Form

ME has been described in the medical literature since 1936 [6]. This was often on account of outbreaks [5,17], the two most well-known being the epidemic in Akureyri district in Iceland (1948–1949) [18] and the outbreak in the Royal Free Hospital (London, UK) in 1955 [15]. However in the 1950s it became evident that ME can also occur in sporadic form [5,16]: “Endemic prevalence alternates with periodic epidemics, showing a curious predilection for female staff of health care and teaching institutions” [3].

#### 3.1.2. An Often Sudden, Sometimes Gradual, Onset

ME often has a sudden onset; in most cases an infection described as a respiratory and/or gastro-intestinal illness, “but a gradual or more dramatic onset following neurological, cardiac or endocrine disability is recognised.” [4]. This implies that a sudden onset is not a distinctive feature of ME as suggested by others [19,20,21].

#### 3.1.3. An Acute and a Chronic Phase

“The onset [..] is usually acute with systemic prodromata such as are common in poliomyelitis.” [7]. In the outbreaks, the prodromal phase was usually followed by improvement for a few hours, days or weeks, after which the symptoms returned more severely with additional symptoms, including marked muscle weakness [1,22].

In many cases the symptoms become chronic with a periodic relapsing course [1].

#### 3.1.4. Not Just Like Other Post-Viral Fatigue States

ME should be distinguished from other forms of “post-viral debility following Epstein–Barr mononucleosis, influenza and other common fevers” [3] since ME has “an unique clinical and epidemiological pattern” [3] and these other post-viral states “lack the dramatic effect of exercise upon muscle function, the multisystem involvement, diurnal variability of symptoms and prolonged relapsing course” [3]. This is also one of the many reasons why ME [1,2,3,4] should not be confused with CFS [11], an ill-defined syndrome which can be initiated by various infections, e.g., the Epstein–Barr virus (mononucleosis), *Coxiella burnetii* (Q fever), or Ross River virus infections [23].

#### 3.1.5. A Prolonged Relapsing Course

A distinctive feature of ME is a prolonged relapsing course. Years can pass between two relapses. Relapses can be caused by physical and mental stress (very often) and intercurrent infection (often). Although outbreak patients appear to recover much often than sporadic patients [22], the illness often lasts for years or even decades.

#### 3.1.6. Diurnal Variability of Symptoms

In addition to periodic relapses or partial remission, ME is characterized by a strong fluctuation of symptoms during the course of the day and over time [1,3]. Physical and/or mental stress can have a (prolonged) negative effect on the symptoms [1].

### 3.2. Symptoms

Although ME is a multi-systemic disease [3] with a large range of symptoms that can vary greatly per patient per day, the distinctive features of ME are limited to muscle fatigability/post-exertional muscle weakness, and specific neurological symptoms.

Patients often report various other symptoms relating to the muscles and “variable involvement of cardiac and other bodily systems” [3], e.g., myalgia (80%), and gastro-intestinal symptoms (49%), but these symptoms are not noted in all cases of ME.

#### 3.2.1. Muscle Fatigability/Prolonged Muscle Weakness after Exertion is a Mandatory Feature of ME

“ME is [..] distinguished by severe muscle fatigue following trivial exertion” [3]. “Muscle fatigability is the dominant and most persistent feature of the disease and [..] a diagnosis should not be made without it. Restoration of muscle power after exertion can take three to five days or even longer.” [1]. This implies that muscle fatigability/prolonged muscle weakness after exertion is a discriminative feature of ME. Long-lasting muscle weakness after exertion can be validated objectively by measuring muscle power of the arms, hands [24,25], legs [26,27], etc., during repeated muscle contractions on two consecutive days using dynamometers [28,29].

#### 3.2.2. Neurological Disturbance Is an Essential Feature of ME

In addition to muscle fatigability/post-exertional muscle weakness, neurological symptoms are distinctive for ME. Neurological disturbance in ME especially relates to “cognitive, autonomic and sensory functions” [1]. Many symptoms observed in epidemic and endemic cases of ME [1] are related to neurological dysfunction. The neurological symptoms relate to cognitive dysfunction (*n* = 3), autonomic dysfunction (*n* = 11), sensory dysfunction (*n* = 19), and motor dysfunction (*n* = 4), while three symptoms (headache and symptoms related to sleep) are unclassified (Table 3). Considering the most recent definitions of ME [3,4] and the frequency of symptoms reported by 420 patients [3], a patient should experience at least one symptom related to cognitive dysfunction (*n* = 3) or one unclassified neurological symptom (*n* = 3), one symptom related to autonomic dysfunction (*n* = 11) and two symptoms related to sensory dysfunction (*n* = 19) to meet the proposed operational definition of ME [1,2,3,4].

#### 3.2.3. ME Often Coincides with a Number of Symptoms Related to Other Body Systems

In addition to the two mandatory features, muscle fatigability/prolonged post-exertional muscle weakness after and neurological dysfunction, ME patients often, but not always, suffer from many other symptoms related to other bodily systems, including musculoskeletal symptoms, e.g., myalgia, paresis (without exercise) and joint pain, immunological symptoms, e.g., sore throat, tender lymph nodes, and lymphadenopathy, gastro-intestinal symptoms, e.g., nausea, anorexia, diarrhoea and abdominal pain, and respiratory symptoms, e.g., respiratory distress, intercostal myalgia (often made worse on breathing) and exertional chest pain with or without palpitations. ME patients can experience “extreme exhaustion”/”gross fatigue” after physical or cognitive exertion and emotional strain [1], but looking at the definitions [2,3,4] and symptoms reported [1,3], the abstract concept of post-exertional “malaise” [11,13], cannot be considered a mandatory feature for the diagnosis of ME [1,2,3,4].

#### 3.2.4. ME Is Often Accompanied by Emotional Problems, but Is Not Caused by Those Symptoms

Emotional lability is a characteristic feature of ME [1,2,3,4]. According to the last patient population study [3], 98% of the 420 ME patients reported experiencing “emotional disability”. Other emotional symptoms reported in cases of ME [4] include “outbursts of irritability“, impatience, anxiety, and depression. However, it should be emphasized that “emotional lability” and other psychological sequels are caused by ME (likely due to cerebral dysfunction), and do not cause the symptoms.

### 3.3. An Operational Definition of ME

Considering the descriptions, definitions [3,4], the symptoms experienced by 420 patients [3], and the symptoms observed in endemic and epidemic cases of ME [1], a discriminative operational definition encompasses four (mandatory) features:

1. muscle fatigability/prolonged muscle weakness after trivial exertion;
2. neurological disturbance (see Table 3):
  - at least one symptom related to cognitive dysfunction or one unclassified neurological symptom;
  - at least one symptom related to autonomic dysfunction; and
  - at least two symptoms related to sensory dysfunction;
3. fluctuation of symptoms (within and between “episodes”); and
4. a prolonged relapsing course.

This definition contains the basic requirements to comply with the diagnosis of ME [3,4]. The tentative case definition described above should be used in conjunction with a full clinical assessment, with consideration of differential diagnoses.

Future surveys should make clear if stricter requirements can be imposed on the three neurological criteria, and if symptoms not mentioned in the original description of endemic and epidemic cases [1] can be added to these neurological symptoms clusters, without excluding patients meeting the minimal requirement for “neurological disturbance”.

Forthcoming studies should also establish the frequency of the (optional) symptoms related to other bodily systems observed in endemic and epidemic cases of ME [1] and mentioned in other diagnostic criteria, e.g., the International Consensus Criteria for ME (ME-ICC) [30], the Fukuda criteria for CFS [11], and the criteria for Systemic Exertion Intolerance Disease (SEID) [13].

Prospective studies should also investigate the overlap and differences between patients meeting the four different systems for diagnoses: ME [1,2,3,4], ME-ICC [30], CFS [11], and SEID [13], and the relative numbers of patients fulfilling one or more of these diagnoses.

Future research should also clarify which signs are exhibited by all patients meeting the operational definition (the “minimum criteria”) of ME and can be included in the definition, and which comorbidities (symptom patterns) are often present in ME.

## 4. Discussion

ME is a distinct neuromuscular disease with specific features [1,2,3,4]. Based on the definitions, ME is not an equivalent of CFS [11], an ill-defined fatigue syndrome. This observation is completely independent of the question whether the name “ME” is appropriate or not [31]. The discussion with regard to the definition (case criteria) should be separated from the discussion with regard to the most accurate label.

Since the cause of ME [1,2,3,4] is yet unknown, the operational definition of ME proposed in this article still depends on self-report of symptoms. However, various symptoms, including muscle fatigability/prolonged muscle weakness (the distinctive symptom) and several neurological symptoms, can be assessed with objective tests.

In 2011 a panel of experts proposed using the International Consensus Criteria (ICC) for ME [30]. The definition of the ME-ICC [30] deviates at different points from the original definition of ME [3,4]. Long-lasting post-exertional muscle weakness is an optional feature of ME according to the ICC [30], but is mandatory for diagnosis according to the original definition [3,4]. On the other hand, diagnosis as per the ME-ICC [30] requires several symptoms which were not part of the original definition of ME [3,4]. Future surveys using the operational definition of ME proposed here should make clear how big the overlap is between the original definition of ME [3,4] and that of the ME-ICC [30] and whether the latter can adequately replace the former according to the original criteria or not.

According to the definition of “ME/CFS” proposed by the Institute of Medicine (now the National Academy of Medicine) in 2015 [13], post-exertional “malaise” is a differential feature of “ME/CFS” (in addition to fatigue and unrefreshing sleep). However, post-exertional “malaise”, an ill-defined notion, has never been described as a distinctive feature of ME [1,2,3,4] in the literature. Long-lasting post-exertional muscle weakness, the distinguishing feature of ME [1,2,3,4], is a very specific symptom which can be assessed objectively and is not a synonym of post-exertional “malaise”.

Other authors have also proposed an operational definition of ME [19,20,21], but this definition has two important shortcomings. The definition [19,20,21] requires a sudden onset. Although ME is often initiated by an infection [2], a gradual onset is also possible [1,2,3,4]. The operational definition proposed [19,20,21] also interprets muscle fatigability/post-exertional muscle weakness as post-exertional “malaise”, but as argued, (post-exertional) muscle weakness and “malaise” are two different concepts.

The tentative definition described here deviates from the London criteria [32] on two crucial points. Exercise-induced fatigue, the first criterion of the London criteria, has never been described a distinctive feature of ME [1,2,3] in the literature, while on the other hand, muscle fatigability/long-lasting post-exertional muscle weakness, the hallmark feature of ME [1,2,3,4], is not required to meet the London criteria [32].

The operational definition of ME also deviates from the definition proposed by Hyde [33], since the proposed operational definition only incorporates discriminative features of ME and not symptoms related to “variable involvement of cardiac and other systems” [3], and does not include “testable brain changes” [33] (which are not proven yet).

## 5. Conclusions

ME is a specific neuromuscular disease [1,2,3,4] and is not equivalent to CFS [11] or “ME/CFS” [13]. ME was described in the literature from 1938 [6] until 1992 [4], after which CFS [11], a fatigue syndrome, pushed ME [1,2,3,4] to the background.

To unravel the aetiology and pathophysiology and to develop effective therapies for of ME as described in the literature [1,2,3,4], it is essential to investigate patients with ME (and ME only). For that reason, an operational definition is indispensable.

This article proposes an operational definition of ME, based on the last formal definitions of ME [3,4], and the symptoms observed in the epidemic and endemic cases of ME [1] and reported by a large group of patients with well-defined ME [3].

An discriminative operational definition of ME should consists of the four mandatory features (minimum criteria for the diagnosis ME [1,2,3,4]):

1. muscle fatigability/prolonged muscle weakness after trivial exertion;
2. neurological disturbance, especially of cognitive, autonomic and sensory functions;
3. fluctuation of symptoms (within and between ‘episodes’); and
4. a prolonged relapsing course.

The first two criteria of this tentative definition of ME justify the qualification “neuromuscular disease”, with specific characteristic and discriminative symptoms.

Muscle fatigability/post-exertional muscle weakness (criterion 1) should be assessed objectively. This article proposes three criteria to operationalize “neurological disturbance” (criterion 2): at least one symptom related to cognitive dysfunction or one unclassified neurological symptom, one symptom related to autonomic dysfunction, and two symptoms related to sensory dysfunction (listed in Table 3). Several neurological symptoms can be confirmed objectively by medical tests.

The criteria “fluctuation of symptoms” (criterion 3) and a “prolonged relapsing course” (criterion 4) will largely depend on self-report. The notion “prolonged” is not operationalized in the literature. However, since an early diagnosis is crucial, future experiments (surveys) should make clear which minimum duration is applicable.

Based on the (proposed) minimum criteria for ME [1,2,3,4]), which discriminate ME from CFS [11] and other disorders, future studies into the criteria for “neurological disturbance” and the frequency of optional symptoms can be conducted.

## Figures and Tables

**Table 1:** The most recent formal definitions of ME (1990) [3].

Definition of ME (1990)
“A syndrome commonly initiated by respiratory and/or gastro-intestinal infection but an insidious or more dramatic onset following neurological, cardiac or endocrine disability occurs.
The pathognomonic features (of ME) are: Complaint of general or local muscular fatigue following minimal exertion with prolonged recovery time. Neurological disturbance, especially of cognitive, autonomic and sensory functions. Variable involvement of cardiac and other systems. A prolonged relapsing course.
Other characteristics include [..] a prolonged relapsing course and variation in intensity of symptoms within and between episodes, tending to chronicity.”

**Table 2:** The most recent formal definitions of ME (1992) [4].

Definition of ME (1992)
“A syndrome initiated by a viral infection commonly described as a respiratory/gastro intestinal illness but a gradual or more dramatic onset following neurological, cardiac or endocrine disability is recognised. The cardinal features, in a patient who has previously been physically and mentally fit, with a good work record are: Generalised or localised muscle fatigue after minimal exertion with prolonged recovery time. Neurological disturbance, especially of cognitive, autonomic and sensory functions, often accompanied by marked emotional lability and sleep reversal. Variable involvement of cardiac and other bodily systems. An extended relapsing course with a tendency to chronicity. Marked variability of symptoms both within and between episodes.”

**Table 3:** Neurological symptoms of patients observed in endemic and epidemic cases [1,3].

**Cognitive Dysfunction**
▯ Neurocognitive impairment (impaired memory or concentration, impaired ability in calculation, using wrong words, forgetfulness, etc.); ▯ Clumsiness/fumble with simple manoeuvres; and ▯ Mental confusion.
**Autonomic Dysfunction**
▯ Dizziness/giddiness/vertigo (when standing); ▯ Loss of equilibrium/imbalance/unsteadiness; ▯ Postural orthostatic tachycardia (POTS): an abnormal heart rate increase (≥30 beats per minute, or to ≥120 beats per minute) after standing still for a while (can be delayed); ▯ Bladder dysfunction: disturbance of micturition (urination), difficulty in starting micturition, nocturia (wake at night one or more times to urinate), pollakiuria (often urinating small amounts), etc.; ▯ Symptoms related to impaired circulation: cold extremities (hands, feet, etc.), ashen-grey facial pallor, etc.; ▯ Symptoms related to thermoregulation: feeling cold, chills, hypothermia, feelings of feverishness (with/without fever), episodes of extreme sweating, etc.; ▯ Intolerance of extremes of temperature/hypersensitivity to climatic change; ▯ Abnormal dryness of the skin of the extremities (head, arms and feet); ▯ Exfoliation of the skin of extremities (head, arms and feet), followed by atrophy of the skin and subcutaneous tissue; ▯ Brittle nails (onychorrhexis); and ▯ Hypertrichosis (abnormal amount of hair growth over the body).
**Sensory Dysfunction**
▯ Paraesthesia (abnormal sensations such as tingling, tickling, pricking, “pins and needles”, numbness, itching or burning of the skin with no apparent cause); ▯ Hypoesthesia (reduced sense of touch or physical sensation/local numbness); ▯ Hypoalgesia (decreased sensitivity to painful stimuli); ▯ Diminished or absent position sense of hands/fingers and feet/toes; ▯ Diminished or absent vibration sense of hands/fingers and feet/toes; ▯ Hyperesthesia (excessive physical sensitivity, especially of the skin); ▯ Hyperalgesia (increased sensitivity to painful stimuli); ▯ Muscle tenderness: upper and lower limbs (arms and legs, e.g., gastrocnemii); ▯ Muscle tenderness: shoulder girdle, e.g., trapezii; ▯ Muscle tenderness: below the ribs; ▯ Muscle tenderness: abdomen, e.g., abdominal recti; ▯ Blurring of (near) vision; ▯ Diplopia (simultaneous perception of two images of a single object); ▯ Loss of accommodation (depth perception); ▯ Photophobia/defective response to light (sluggish pupils)/light hypersensitivity; ▯ Nystagmus (involuntary eye movements, “dancing eyes”); ▯ Hyperacusis (intolerance of loud noise); ▯ Tinnitus; and ▯ Deafness.
**Motor Dysfunction**
▯ Complete paralysis; ▯ Paralysis of the limbs (arms and legs) ▯ Paralysis of the face; ▯ Paralysis of swallowing; ▯ Poor movement of the soft palate; ▯ Muscle spasms (often in limbs); ▯ Muscle twitches/jerking of the limbs/myoclonus; ▯ Fasciculation of muscles (local, involuntary muscle contraction and relaxation); ▯ Exaggerated tendon reflexes of the upper and lower limbs (increase in amplitude and duration of muscle contraction in response to an impulse); ▯ Depressed tendon reflexes of the upper and lower limbs (decrease in amplitude and duration of muscle contraction in response to an impulse); ▯ Extensor plantar responses (Babinski reflex); ▯ Ankle clonus (series of involuntary, rhythmic, muscular contractions and relaxations usually initiated by a reflex); ▯ Ataxia (lack of coordination of muscle movements, including gait abnormality); and ▯ Tendency to stumble when walking, unsteady gait.
**Unclassified Neurological Symptoms**
▯ Reversal of sleep rhythm (not sleepy at night, sleeping in the day); ▯ Vivid dreams/nightmares (often in colour); and ▯ Headaches (often frontal or occipital, sometimes accentuated by movement).
